# CRISPR-Cas9 Mediated RNase L Knockout Regulates Cellular Function of PK-15 Cells and Increases PRV Replication

**DOI:** 10.1155/2019/7398208

**Published:** 2019-03-03

**Authors:** Chao Sui, Dandan Jiang, Xiangju Wu, Xiaoyan Cong, Feng Li, Yingli Shang, Jinqiu Wang, Sidang Liu, Hu Shan, Jing Qi, Yijun Du

**Affiliations:** ^1^Shandong Provincial Key Laboratory of Animal Biotechnology and Disease Control and Prevention, College of Animal Science and Technology, Shandong Agricultural University, Tai'an 271018, China; ^2^Shandong Key Laboratory of Animal Disease Control and Breeding, Institute of Animal Science and Veterinary Medicine, Shandong Academy of Agricultural Sciences, Sangyuan Road No. 8, Jinan 250100, China; ^3^Department of Biology and Microbiology, Department of Veterinary and Biomedical Sciences, South Dakota State University, Brookings, SD 57007, USA; ^4^Department of Animal Husbandry and Veterinary Medicine, Beijing Vocational College of Agriculture, 5 Daotiannanli Road, Beijing 102442, China; ^5^College of Veterinary Medicine, Qingdao Agricultural University, Qingdao 266109, China; ^6^College of Life Science, Shandong Normal University, Jinan 250014, China

## Abstract

Ribonuclease L (RNase L) is an important antiviral endoribonuclease regulated by type I IFN. RNase L is activated by viral infection and dsRNA. Because the role of swine RNase L (sRNase L) is not fully understood, in this study, we generated a sRNase L knockout PK-15 (KO-PK) cell line through the CRISPR/Cas9 gene editing system to evaluate the function of sRNase L. After transfection with CRISPR-Cas9 followed by selection using puromycin, sRNase L knockout in PK-15 cells was further validated by agarose gel electrophoresis, DNA sequencing, and Western blotting. The sRNase L KO-PK cells failed to trigger RNA degradation and induced less apoptosis than the parental PK-15 cells after transfected with poly (I: C). Furthermore, the levels of ISGs mRNA in sRNase L KO-PK cells were higher than those in the parental PK-15 cells after treated with poly (I: C). Finally, both wild type and attenuated pseudorabies viruses (PRV) replicated more efficiently in sRNase L KO-PK cells than the parental PK-15 cells. Taken together, these findings suggest that sRNase L has multiple biological functions including cellular single-stranded RNA degradation, induction of apoptosis, downregulation of transcript levels of ISGs, and antiviral activity against PRV. The sRNase L KO-PK cell line will be a valuable tool for studying functions of sRNase L as well as for producing PRV attenuated vaccine.

## 1. Introduction

The type I interferons (IFNs), consisting of IFN-*α* and IFN-*β*, are critical cytokines used for communication between cells and stimulate protective defenses of the immune system against viral infections. The type I IFNs bind to cells expressing IFN-*α*/*β* receptor (IFNAR) 1 and IFNAR2 and subsequently activate Janus kinase 1 (JAK1) and tyrosine kinase 2 (Tyk2). The signal transducers and activators of transcription 1 (STAT1) and STAT2 heterodimerize upon JAK1/Tyk2-mediated tyrosine phosphorylation and further recruit IFN regulatory factor 9 (IRF9) in the cytoplasm to form a transcriptional activator complex, interferon-stimulated gene factor 3 (ISGF3). ISGF3 then translocates into the nucleus and sequence-specifically binds to an IFN-stimulated response element (ISRE), which boosts the transcription of a number of type I IFN-stimulated genes (ISGs) to initiate the antiviral state. These ISGs include myxovirus resistance 1 (Mx1), double-stranded RNA-dependent protein kinase R (PKR), IFN-stimulated gene 15 (ISG15), 2',5'-oligoadenylate synthetase (OAS), and so on [1–5].

The OAS/RNase L system is one of the early recognized IFN effector pathways [6–9]. The presence of double strand RNA (dsRNA) activates OAS, which in return initiates the synthesis of short oligonucleotides, 2',5'-linked oligoadenylates (2-5A) that act as second messengers to activate the latent cellular RNase L [10]. RNase L is an endoribonuclease with a ubiquitous expression in all mammalian animals and it contains three major domains: an N-terminal regulatory ankyrin repeat domain (ARD), a protein kinase (PK)-like domain, and a C-terminal ribonuclease domain (RNASE) [6]. Active RNase L destroys both viral and cellular RNAs (mRNA and rRNA) within the cells [7, 8]. The degradation of 28S and 18S rRNA is associated with suppressed viral proteins synthesis [9]. In addition, activation of RNase L induces apoptosis in virus-infected cells through the mitochondrial pathway to limit viral spread [11]. Antiviral effects of RNase L have been extensively illustrated in RNA viruses, while its roles in counteracting DNA viruses have been rarely studied.

Pseudorabies virus (PRV) is a member of the* Alphaherpesvirinae* subfamily within the family* Herpesviridae*, which contains a double-stranded DNA genome and causes great economic losses in swine industry [12, 13]. At present, the widely used PRV vaccine has played an important role in the control of pseudorabies (PR). The major vaccines are gE knockout strains, including Bartha-K61 strain [14].

Porcine kidney epithelial cell line PK-15 has been used to study the infections of numerous of porcine viruses, including classical swine fever virus (CSFV), porcine circovirus type 2 (PCV2), and foot-and-mouth disease (FMDV) [15, 16]. Moreover, this cell line has been extensively employed to develop and prepare swine vaccines, such as CSFV C strain and chimeric PCV2 vaccine [17–19]. To study the antiviral role of RNase L, in this study, we engineered and constructed a sRNase L knockout- (KO-) PK cell line by taking the advantage of CRISPR-Cas9 gene editing system. sRNase L KO-PK cells attenuated cellular RNA degradation and inhibited poly (I: C)-induced apoptosis. In addition, the mRNA expression of ISGs in sRNase L KO-PK cells was higher than the wild type. Finally, sRNase L KO-PK cells were more susceptible to PRV in comparison with the parental PK-15 cells. Taken together, our results demonstrated the antiviral activities of sRNase L against PRV infection and suggested the potential benefits of using sRNase L KO-PK cells for vaccine development.

## 2. Materials and Methods

### 2.1. Cells, Virus, and Chemicals

Porcine kidney (PK-15) cells were maintained in Dulbecco's modified Eagle's medium (DMEM) containing 10% fetal bovine serum (FBS; Gibco, Grand Island, USA), penicillin (100 U/mL), and streptomycin (100 *μ*g/mL) in a humidified incubator with 5% CO_2_ at 37°C. PRV SD1404 strain was propagated and titrated in PK-15 cells and stored at -80°C in our lab. The complete gE, gD, TK, and gM gene sequences of PRV SD1404 strain were deposited in GenBank under the accession numbers KP315914, KP315913, MG581434, and MG581433. PRV Bartha-K61 vaccine strain was obtained from Nanjing Tianbang Bio-industry Co., Ltd. Poly (I: C) (Sigma-Aldrich, St. Louis, MO, USA), a surrogate for viral double-stranded RNA (dsRNA), was used to activate the OAS/RNase L pathway.

### 2.2. Construction of Px459M-sRNase L-KO Plasmid

**Figure 1:**
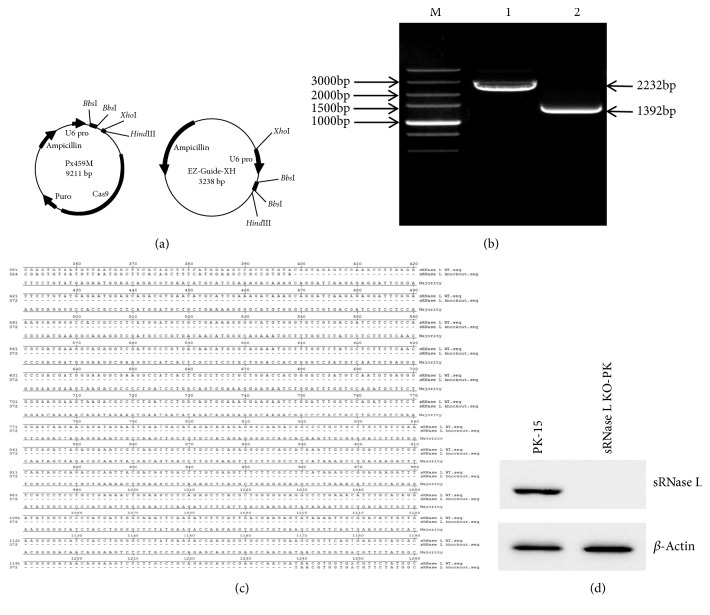
Generation and identification of sRNase L KO-PK cell line. (a) The plasmid map of Px459M and EZ-Guide-XH used to generate sRNase L KO-PK cell line. (b) The total RNAs of PK-15 and sRNase L KO-PK cells were extracted and analyzed by RT-PCR using PrimerScript One step RT-PCR Kit on 1% agarose gel. Lane 1, parental PK-15 cells (2232 bp band). Lane 2, sRNase L KO-PK cells (1392 bp). (c) RT-PCR product of the correct clone was sequenced and compared with the sRNase L gene sequence of PK-15 cells. (d) Western blotting analysis of sRNase L KO-PK cells. PK-15 and sRNase L KO-PK cells were seeded in 6-well plates for 16 h and harvested for Western blotting with anti-sRNase L antibody. The same blot was incubated by *β*-actin antibody as a protein loading control. The data showed here were results from one experiment of three Western blotting experiments.

Specific gene-targeted sgRNAs of sRNase L were designed using an online CRISPR Design Tool (http://crispr.mit.edu/). Two sgRNAs for editing sRNase L were used in this study and listed in Table 1. The sRNase L-KO-3-Fwd and sRNase L-KO-3-Rev were annealed to form sRNase L-KO-3P and inserted into Px459M (Px459 pSpCas9-2A-Puro-MCS) vector (Figure 1(a)) by* Bbs*I site. The sRNase L-KO-4-Fwd and sRNase L-KO-4-Rev were annealed to form sRNase L-KO-4P and inserted into EZ-Guide-XH vector (Figure 1(a)) by* Bbs*I site. And then, sRNase L-KO-4P was inserted into Px459M vector by* Xho*I and* Hin*dIII sites from EZ-Guide-XH vector to form Px459M-sRNase L-KO. The plasmids were sequenced to confirm the correct insertion of sRNase L-KO-3P and sRNase L-KO-4P.

### 2.3. Generation of sRNase L KO-PK Cell Line

PK-15 cells were transfected with Px459M-sRNase L-KO using the Lipofectamine® 3000 reagent (Invitrogen, Carlsbad, CA, USA) according to the manufacturer's instructions. At 24 h after transfection, cells were selected with 5 *μ*g/mL of puromycin (Solarbio, Beijing, China) which was diluted in DMEM with 10% FBS. Three days later, the positive clones were isolated, trypsinized and diluted in 96-well plates. The single cell clones were further digested to culture in 24-well plates. Then half of individual cell clones were used to extract RNA by TRIzol reagent according to the manufacturer's protocol (Invitrogen, Carlsbad, CA, USA) and screened by RT-PCR using PrimerScript One step RT-PCR Kit (Takara, Tokyo, Japan). The RT-PCR primers of sRNase L-Fwd and sRNase L-Rev used in this study were listed in Table 1. RT-PCR products of the correct clones were further performed DNA sequencing analysis.

### 2.4. Production of Polyclonal Antibodies against sRNase L

The sRNase L gene was cloned into pGEX-4T-1 vector which contains a fusion GST tag for purification. After expression, the GST label of GST-sRNase L fusion protein was removed by rTEV protease (Solarbio, Beijing, China). 100 *μ*g purified protein was mixed with Freund's adjuvant and injected intramuscularly into two rabbits. The other two injections were made every two weeks using 100 *μ*g purified protein mixed with Freund's incomplete adjuvant. The antibody titer against sRNase L of the two rabbits was detected by iELISA using the purified sRNase L as coated antigen. The serum with the higher antibody titer was used for further Western blotting analysis.

### 2.5. Western Blotting Assay

PK-15 and sRNase L KO-PK cells were grown in 6-well plates for 16 h and lysed in ice-cold cell lysis buffer supplemented with phenylmethylsulfonyl fluoride (PMSF; Beyotime, Shanghai, China). Samples of cell lysates were analyzed by sodium dodecyl sulfate polyacrylamide gel electrophoresis (SDS-PAGE) and Western blotting. Briefly, the samples were resolved in a 12% polyacrylamide gel. Separated proteins were then transferred onto a nitrocellulose membrane and probed with anti-sRNase L antibody or *β*-actin antibody (Solarbio, Beijing, China). Specific reaction products were detected with horseradish peroxidase- (HRP-) conjugated goat anti-rabbit IgG or goat anti-mouse IgG (Boster, Wuhan, China). The membranes were developed using SuperSignal West Pico Chemiluminescent Substrate according to the manufacturer's suggestions (Pierce, Rockford, IL, USA). Digital signal acquisition and analysis were conducted by the Quantity One program, version 4.6 (Bio-Rad).

### 2.6. rRNA Degradation Assay

PK-15 and sRNase L KO-PK cells were seeded in 12-well plates and grown to 70 to 80% confluence. Cells were transfected with 2 *μ*g/mL poly (I: C) for 7 h. The untransfected cells were used as negative control. The total RNA was extracted using TRIzol reagent according to the manufacturer's protocol. rRNA degradation was assessed by running RNA samples on 1% agarose gels in Tris-acetate-EDTA (TAE) buffer.

### 2.7. TUNEL Apoptosis Assay

PK-15 and sRNase L KO-PK cells were directly seeded onto coverslips in 24-well plates and transfected with 2 *μ*g/mL poly (I: C) after cultured overnight. At 7 h after transfection, cells were washed twice in ice-cold PBS and fixed with 4% paraformaldehyde in PBS at 4°C for 1 h. After washed three times with ice-cold PBS, cells were permeabilized with 0.5% Triton X-100 in PBS for 15 min. And then, the coverslips were labeled using One Step TUNEL apoptosis assay kit according to the manufacturer's protocol (Beyotime, Shanghai, China). The coverslips were treated with 4',6'-diamidino-2-phenylindole (DAPI; Sigma-Aldrich, St. Louis, MO, USA) to view the nuclei. After three washes, the coverslips were mounted with antifade mounting medium (Solarbio, Beijing, China) and observed under an Olympus BX51 inverted fluorescence microscope. The number of cells measuring of apoptosis was determined by counting 100 cells each in random microscopic fields. Each experiment was conducted in triplicate and repeated three times.

### 2.8. Growth Kinetics

The PK-15 and sRNase L KO-PK cells were grown in 12-well plates to 80% confluence and infected with PRV SD1404 or Bartha-K61 vaccine strain at the same multiplicity of infection (MOI) of 1. At 12, 24, 36, 48, and 60 h after infection, the supernatants were collected. One part of culture supernatants was titrated by a microtitration infectivity assay on PK-15 cells. The 50% tissue culture infective dose (TCID_50_) was calculated by the Reed-Muench method [20]. The remaining part of culture supernatants was analyzed using real-time PCR assay. All assays were repeated at least three times, with each experiment performed in triplicate.

### 2.9. Real-Time PCR

To test the effect of sRNase L on the transcript levels of ISGs, parental PK-15 and sRNase L KO-PK cells were grown in 12-well plates to 70 to 80% confluence. Cells were transfected with 2 *μ*g/mL poly (I: C) for 7 h. The total RNA was extracted using TRIzol reagent (Invitrogen, Carlsbad, CA, USA). First-strand cDNA was synthesized using PrimeScript RT reagent kit with gDNA Eraser (TaKaRa, Tokyo, Japan) and 1 *μ*l cDNA was subsequently used for SYBR green PCR assay (TaKaRa, Tokyo, Japan). Real-time PCR primers used in this study were listed in Table 1. The abundances of individual mRNA transcripts in each sample were assayed three times and normalized to the level of porcine glyceraldehyde-3-phosphate dehydrogenase (GAPDH) mRNA (as an internal control). Relative transcript levels were quantified by the 2^-∆∆CT^ (where C_T_ is threshold cycle) method and shown as fold change relative to the level for the mock-treated control untransfected cells.

Real-time PCR was also performed to evaluate PRV DNA level. Viral DNA of culture supernatants was extracted by using DNAzol reagent (Invitrogen, Carlsbad, CA, USA) following the instructions of the manufacturer. The primers used for PRV gD gene amplification were listed in Table 1. The DNA of PRV SD1404 strain was tenfold serially diluted and used to generate the standard curve. Relative PRV DNA levels of samples were determined by linear extrapolation of the C_T_ value plotted against the standard curve. All assays were repeated at least three times, with each experiment performed in triplicate.

### 2.10. Statistical Analysis

Data were compared and the differences were determined by one-way repeated measurement ANOVA and least significance difference (LSD). A* P*-value < 0.05 was considered statistically significant [21].

## 3. Results

### 3.1. Construction of sRNase L KO-PK Cell Line Using the CRISPR-Cas9 Gene Editing System

The oligonucleotides for the guide RNA were annealed and inserted into Px459M vector to generate the plasmid Px459M-sRNase L-KO. The plasmid Px459M-sRNase L-KO was then transfected into PK-15 cells. After puromycin selection, positive clones were isolated, trypsinized and diluted in 96-well plates. Cell clone candidates were identified and confirmed by RT-PCR, DNA sequencing, and Western blotting. Compared to parental PK-15 cells (2232 bp band, lane 1), RT-PCR showed a 1392 bp band in sRNase L KO-PK cells (lane 2), which is suggestive of the deletion of sRNase L sequence (Figure 1(b)). DNA sequencing further confirmed that 399-1238 bp of sRNase L gene was knocked out in sRNase L KO-PK cells (Figure 1(c)). There was no sRNase L protein expression in sRNase L KO-PK cells by Western blotting (Figure 1(d)).

### 3.2. sRNase L Mediates rRNA Degradation

**Figure 2:**
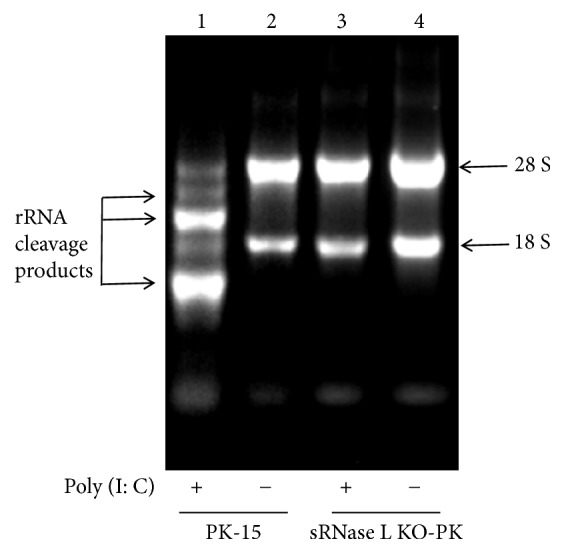
The rRNA degradation in PK-15 and sRNase L KO-PK cells. PK-15 and sRNase L KO-PK cells were transfected or mock-transfected with poly (I: C). At 7 h after transfection, total cellular RNA was extracted and analyzed on 1% agarose gel.

Poly (I: C) has been used to mimic viral dsRNA-induced OAS/RNase L pathway [8, 22]. To determine whether sRNase L knockout blocks rRNA degradation, both parental PK-15 and sRNase L KO-PK cells were transfected with 2 *μ*g/mL of poly (I: C) for 7 h, and then the total cellular RNA was extracted and analyzed on 1% agarose gels in TAE buffer. The rRNA degradation was observed in poly (I: C)-treated PK-15 cells (Figure 2, lane 1), but not in sRNase L KO-PK cells (Figure 2, lane 3). No rRNA degradation appeared in mock-treated cells (Figure 2, lanes 2 and 4). These results suggested that sRNase L induced cellular rRNA degradation after activated by poly (I: C).

### 3.3. sRNase L Is Indispensable for Poly (I: C)-Induced Cell Apoptosis

**Figure 3:**
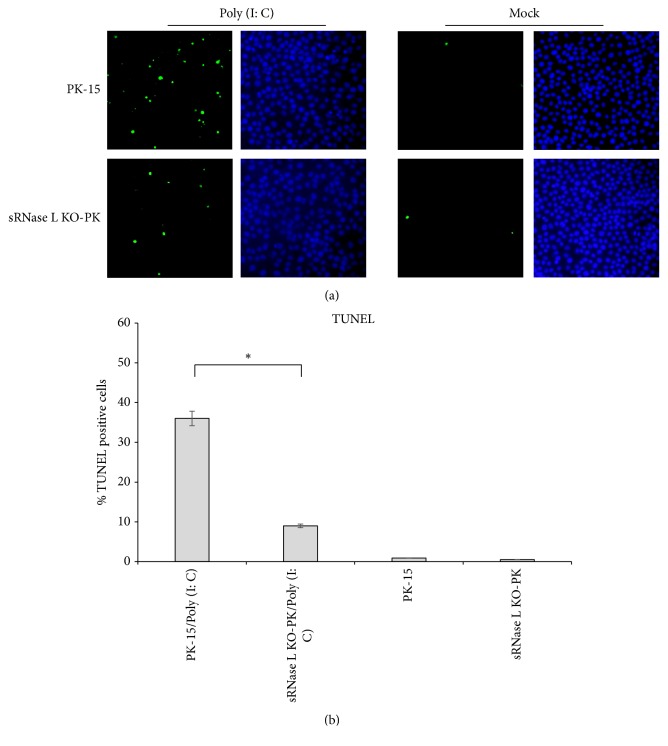
Apoptosis in poly (I: C) transfected PK-15 and sRNase L KO-PK cells. (a) The nicked DNA in apoptotic cells was stained with green color by TUNEL assay and the nuclei were stained with blue color by DAPI. (b) The percentage of TUNEL-positive cells. The number of cells measuring of apoptosis was determined by counting 100 cells each in random microscopic fields. Each experiment was conducted in triplicate and repeated three times. Error bars indicate the standard deviations of three experiments. *∗*,* P* < 0.05.

To address if sRNase L is required for viral-induced apoptosis, parental PK-15 and sRNase L KO-PK cells were transfected with 2 *μ*g/mL poly (I: C) for 7 h and subjected to TUNEL staining. The number of apoptotic cells of PK-15 cells was significantly higher than sRNase L KO-PK cells (*P* < 0.05) following poly (I: C) treatment. No significant difference was observed between these two cells in the absence of poly (I: C) (Figures 3(a) and 3(b)). These results suggested the essential role of sRNase L in poly (I: C)-induced apoptosis.

### 3.4. sRNase L Interferes with ISGs mRNA Synthesis

**Figure 4:**
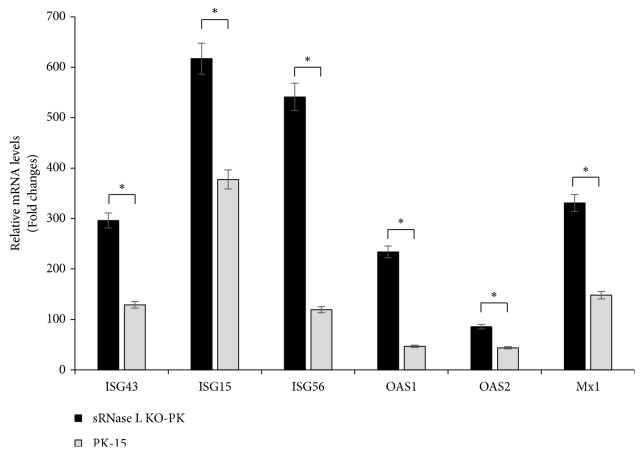
RNase L reduces the transcript levels of ISGs. PK-15 and sRNase L KO-PK cells were transfected with poly (I: C). 7 h later, the transcript levels of ISG43, ISG15, ISG56, OAS1, OAS2, and Mx1 were analyzed by real-time RT-PCR. The cells without poly (I: C) treatment were used as negative controls and GAPDH was used as an internal control. The data represent the means of three independent experiments, with each experiment performed in triplicate. Data represent the means ± the standard deviations (error bars) of three experiments. *∗*,* P* < 0.05.

Since the activation of type I IFN signaling pathway leads to the expression of over 300 ISGs [23, 24], we examined mRNA expression of ISG43, ISG15, ISG56, OAS1, OAS2, and Mx1 in PK-15 and sRNase L KO-PK cells by real-time RT-PCR following poly (I: C) transfection. sRNase L KO-PK cells showed significantly increased expression of all examined genes in comparison with parental PK-15 cells (*P* < 0.05) (Figure 4), suggesting a negative regulatory role of sRNase L in IFN-stimulated responses.

### 3.5. sRNase L KO-PK Cells Are Highly Susceptible to PRV Wild Type and Vaccine Strains

**Figure 5:**
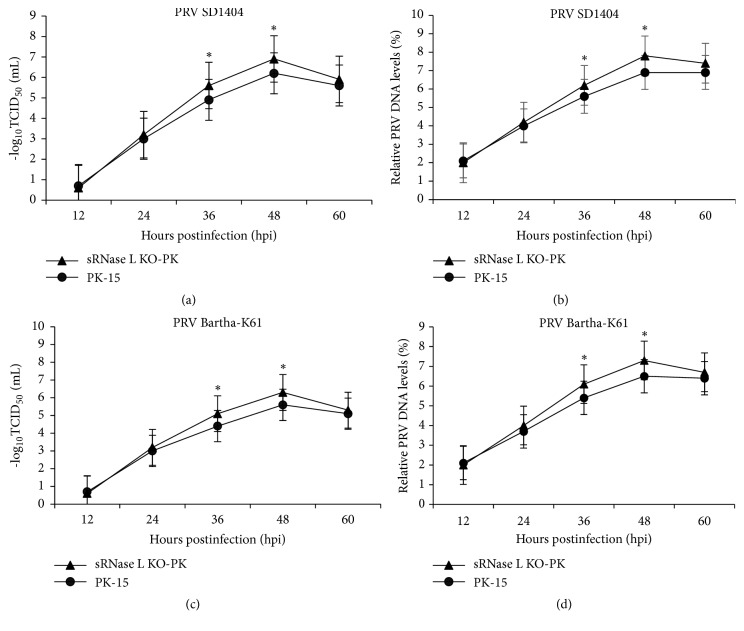
Growth kinetics of PRV in PK-15 and sRNase L KO-PK cells. PK-15 and sRNase L KO-PK cells were individually infected with PRV SD1404 or Bartha-K61 vaccine strain at an MOI of 1. Culture supernatants were collected at the indicated times. The virus titers of PRV SD1404 (a) or Bartha-K61 (c) strain were determined. The relative PRV DNA levels of PRV SD1404 (b) or Bartha-K61 (d) strain were analyzed by real-time PCR. The data represent the means of three independent experiments, with each experiment performed in triplicate. Error bars indicate the standard deviations of three experiments. *∗*,* P* < 0.05.

To determine the susceptibility of sRNase L KO-PK cells to PRV, sRNase L KO-PK and parental PK-15 cells were infected with PRV SD1404 or Bartha-K61 vaccine strain at an MOI of 1. Single-step growth experiments were performed and viral infectivity (TCID_50_ per mL) was measured over time (Figures 5(a) and 5(c)). Relative PRV DNA levels were also analyzed by real-time PCR (Figures 5(b) and 5(d)). PRV SD1404 or Bartha-K61 vaccine strain replicated more efficiently in sRNase L KO-PK cells than parental PK-15 cells (*P* < 0.05). The virus titers produced by sRNase L KO-PK and PK-15 cells for PRV SD1404 or Bartha-K61 vaccine strain at 12, 24, 36, 48, and 60 h after infection were listed in Table 2. The titers of PRV SD1404 or Bartha-K61 vaccine strain in sRNase L KO-PK cells were significantly higher than those in PK-15 cells at 36 h and 48 h after infection (*P* < 0.05). It was shown that sRNase L KO-PK cells were more susceptible to infections by PRV wild type and vaccine strains. In sum, the sRNase L KO-PK cell line could be used toward improving virus yield during vaccine production.

## 4. Discussion

The CRISPR-Cas9 technique is simple, reliable, and inexpensive genome editing tool in comparison with other earlier methods, such as homologous recombination (HR), zinc-finger nucleases (ZFNs), and transcription activator-like effector nucleases (TALENs) [25, 26]. The CRISPR-Cas9 gene editing system facilitates the studies on the biological function of cellular proteins due to its convenience to construct gene knockout cell lines. In this study, we developed a sRNase L KO-PK cell line by deleting sRNase L gene using the CRISPR-Cas9 gene editing system, and determined antiviral role of sRNase L.

We used poly (I: C) to induce the activation of sRNase L through OAS/RNase L pathway [27]. Active sRNase L manipulates virus replication through cleaving cellular and viral RNA [28] and inducing cell apoptosis [29]. In this study, rRNA was degraded in parental PK-15 cells but not in sRNase L KO-PK cells (Figure 2). Since the host cellular organelles such as ribosomes are indispensable for the synthesis of viral protein and thereafter the viral replication, it is plausible that sRNase L-induced rRNA degradation suppresses virus replication. In addition, sRNase L expression had been associated with the activation of apoptosis signaling which led to cellular damage and as a result inhibited viral replication. Being consistent with previous reports [29], we also showed significantly increased number of apoptotic PK-15 cells compared to sRNase L KO-PK cells following poly (I: C) stimulation (Figure 3).

Type I IFN signaling and subsequent expression of ISGs are strictly regulated to avoid deleterious effects due to ISG overexpression. A previous study has suggested that the mRNA expression of ISG15 and ISG43 negatively regulated by RNase L in an RNase L-deficient murine neuroblastoma cell line [30]. Moreover, an RNase L-mediated negative regulation of PKR mRNA expression was noticed in RNase L^−/−^ MEFs [31, 32]. In this study, we found that sRNase L exclusively downregulates all examined ISG mRNA expressions including ISG43, ISG15, ISG56, OAS1, OAS2, and Mx1 (Figure 4), which is consistent with previous studies [30].

The antiviral effects of RNase L have been described on Encephalomyocarditis virus (EMCV), Coxsackie B4 virus, West Nile virus, Coronaviruses, Herpes Simplex virus-1 (HSV-1), and Vaccinia virus (VV) [33–40]. Recent studies have indicated several mechanisms of RNase L-mediated antiviral activity. For instance, RNase L cleaves hepatitis C virus (HCV) genome into 200-500 bp fragments at UU and UA dinucleotides [41]. Filamin A, a multifunctional actin-binding protein, interacts and cooperates with RNase L to block virus entry [42]. The current study demonstrated that sRNase L expression inhibited PRV replication in PK-15 cells (Figure 5). Attenuated PRV vaccines, in particular the PRV Bartha-K61 strain, have been widely used by swine industry to control pseudorabies [14]. In this study, sRNase L KO-PK cells showed increased replication of PRV Bartha-K61 vaccine strain than parental PK-15 cells (Figures 5(c), and 5(d) and Table 2). Hence, the attenuated antiviral activity in sRNase L KO-PK cells makes it a useful cell line to grow viruses. The sRNase L KO-PK cell line may have the potential for use in production of attenuated vaccines of other viruses that can cause a significant concern to animal health industry.

## 5. Conclusions

We have successfully developed a sRNase L KO-PK cell line with sRNase L gene deleted through the CRISPR-Cas9 genome editing system. We further applied this edited cell line to evaluate multiple biological roles of sRNase L and its antiviral activity against PRV infection. The sRNase L KO-PK cell line should be a valuable tool to study biological functions of sRNase L and its full-spectrum of antiviral activities. This sRNase L KO-PK cell line can be used as a candidate cell line to optimize PRV vaccine production.

## Figures and Tables

**Table 1:** Primers used in this study.

Primer name	Sequence (5'-3')	Purpose
sRNase L-KO-3-Fwd	CACCTTCATGGAAGCCGCCGTGTA	sRNase L knockout
sRNase L-KO-3-Rev	AAACTACACGGCGGCTTCCATGAA	
sRNase L-KO-4-Fwd	CACCCAGCCGAGCCAACGATAACG	sRNase L knockout
sRNase L-KO-4-Rev	AAACCGTTATCGTTGGCTCGGCTG	
sRNase L-Fwd	ATGGAGACCAAGCGCCATAACAAC	sRNase L amplification
sRNase L-Rev	CTAGGTCTGGCCATCACCAGCTC	
sISG43-Fwd	TGAGGAGCAGAGGAGAAATG	ISG43 amplification
sISG43-Rev	GGAGACAGTAGGCAAGTTCC	
sISG15-Fwd	GCAATGTGCTTCAGGATGG	ISG15 amplification
sISG15-Rev	AGGCTTGAGGTCATACTCCC	
sISG56-Fwd	GGAGTTGGTCATTCAAGACAC	ISG56 amplification
sISG56-Rev	CGTAAGGTAATACAGCCAGGC	
sOAS1-Fwd	ATGCTGACCTCGTCGTCTTC	OAS1 amplification
sOAS1-Rev	GGACATCAAACTCCACCTCC	
sOAS2-Fwd	TCTGGGCACAGTTGAAATG	OAS2 amplification
sOAS2-Rev	GATGCTCTGCTCTTTAGCG	
sMx1-Fwd	TGAACGAAGAAGACGAATGG	Mx1 amplification
sMx1-Rev	CGTATGGCTGATTGCCTAC	
sGAPDH-Fwd	ATCACCATCTTCCAGGAGC	GAPDH amplification
sGAPDH-Rev	TTCACGCCCATCACAAAC	
PRV-Fwd	TGAACATCCTCACCGACTTC	PRV gD amplification
PRV-Rev	TAGAACGGCGTCAGGAATC	

**Table 2:** Ability of sRNase L KO-PK and PK-15 cells for PRV propagation.

PRV strain	Harvest time	Virus titers in sRNase L KO-PK cells ^a^	Virus titers in PK-15 cells ^a^	Fold-difference in PRV titers ^b^
SD1404	12 h	1×10 ^0.6±0.1^ TCID_50_/mL	1×10 ^0.7±0.2^ TCID_50_/mL	0.79
Bartha-K61	12 h	1×10 ^0.6±0.2^ TCID_50_/mL	1×10 ^0.7±0.3^ TCID_50_/mL	0.79
SD1404	24 h	1×10 ^3.2±0.1^ TCID_50_/mL	1×10 ^3.0±0.3^ TCID_50_/mL	1.58
Bartha-K61	24 h	1×10 ^3.1±0.3^ TCID_50_/mL	1×10 ^2.9±0.1^ TCID_50_/mL	1.58
SD1404	36 h	1×10 ^5.6±0.2^ TCID_50_/mL*∗*	1×10 ^4.9±0.2^ TCID_50_/mL	5.01
Bartha-K61	36 h	1×10 ^5.1±0.1^ TCID_50_/mL*∗*	1×10 ^4.4±0.2^ TCID_50_/mL	5.01
SD1404	48 h	1×10 ^6.9±0.2^ TCID_50_/mL*∗*	1×10 ^6.2±0.1^ TCID_50_/mL	5.01
Bartha-K61	48 h	1×10 ^6.4±0.2^ TCID_50_/mL*∗*	1×10 ^5.6±0.3^ TCID_50_/mL	6.31
SD1404	60 h	1×10 ^5.9±0.3^ TCID_50_/mL	1×10 ^5.4±0.1^ TCID_50_/mL	3.16
Bartha-K61	60 h	1×10 ^5.3±0.1^ TCID_50_/mL	1×10 ^5.1±0.2^ TCID_50_/mL	1.58

^a^Data represent the means ± standard deviations of three independent experiments. *∗*, *P *< 0.05.

^b^Fold-difference in PRV titers was obtained by PRV titers in sRNase L KO-PK cells/PRV titers in PK-15 cells.

## Data Availability

The data used to support the findings of this study are included within the article.
